# Case Report: Bilateral Deep Brain Stimulation Implantation on Different Targets for a Parkinson's Disease Patient With a Bullet in the Brain

**DOI:** 10.3389/fnhum.2021.808231

**Published:** 2022-01-06

**Authors:** Yu Tian, Jiaming Wang, Xin Shi, Zhaohai Feng, Lei Jiang, Yujun Hao

**Affiliations:** Neurosurgery Department, The First Affiliated Hospital of Xinjiang Medical University, Ürümqi, China

**Keywords:** different targets, deep brain stimulation, Parkinson's disease, bullet, case report

## Abstract

Patients requiring deep brain stimulation due to intracerebral metallic foreign substances have not been reported elsewhere in the world. Additionally, the long-term effects of metallic foreign bodies on deep brain stimulation (DBS) are unknown. A 79-year-old man with a 5-year history of Parkinson's disease (PD) reported that, 40 years ago, while playing with a pistol, a metallic bullet was accidentally discharged into the left brain through the edge of the left eye, causing no discomfort other than blurry vision in the left eye. DBS was performed due to the short duration of efficacy for oral medication. Because the bullet was on the left subthalamic nucleus (STN) electrode trajectory and the patient's right limb was primarily stiff, the patient received globus pallidus interna (GPi)-DBS implantation in the left hemisphere and STN-DBS implantation in the right hemisphere. During a 6-month postoperative follow-up, the patient's PD symptoms were effectively managed with no noticeable discomfort.

## Introduction

Parkinson's disease is a common chronic neurodegenerative disorder that primarily affects the motor nervous system of the central nervous system. Its symptoms typically manifest gradually with resting tremor, myotonia, progressive motor decline, and gait abnormalities being the most prominent early symptoms. Additionally, cognitive decline and psychiatric symptoms may manifest. Recent research has indicated that the probability of developing PD is increased in older persons following traumatic brain injury (TBI) and that chronic TBI may be an independent risk factor in PD (Gardner et al., 2015).

Deep Brain Stimulation is an effective treatment for certain neuropsychiatric disorders. It entails implanting electrodes into specific target sites in the brain to electrically stimulate specific nuclei or functional areas. Additionally, deep brain stimulation (DBS) is a critical technological tool for studying brain function and neural pathways. At present, there is no consensus on the ideal target for DBS treatment of PD. According to available research, the subthalamic nucleus (STN) and globus pallidus interna (GPi) are viable targets for PD, and both can improve motor functions and daily life of patients with PD. Both STN and GPi, however, have several advantages and disadvantages. Current clinical practice indicates that bilateral multi-nucleus combination DBS can alleviate symptoms in patients with PD who have asymmetric symptoms.

Herein, we report a case of bilateral different targets of DBS surgical treatment of an elderly patient with PD with residual metal shrapnel in the brain from a gunshot wound sustained in his youth. To the best of our knowledge, DBS has not been reported in patients with retained intracerebral metallic foreign bodies anywhere in the world. Furthermore, the long-term effects of metallic foreign bodies on DBS remain unclear, as is the question of whether brain trauma suffered in youth is associated with the development of PD later in life. In addition, there are challenges in the patient evaluation and examination in terms of symptom assessment, target calculation, and postoperative programming.

## Case Presentation

### Presentation and Examination

A 79-year-old male was admitted to the hospital presenting with resting tremor of the left limb that appeared 5 years ago with no apparent cause, followed by stiffness and weakness of the extremities, resulting in clumsy walking and slow movement. The patient's condition worsened in the last 2 years. Nervous system examination revealed hypertonia in all limbs with brisk tendon reflexes. Resting tremor in all of limbs to varying degrees, the left side is typically more severely affected than the right side. In addition, the patient can independently stand with head and trunk leaning forward, small step, bradykinesia, and positive posterior pull test. He was clinically diagnosed with Parkinson's disease and was treated with Madopar 125 mg q.i.d. and Sifrol 0.25 mg t.i.d. He had a baseline Hoehn-Yahr (H-Y) score of III, and Unified Parkinson's Disease Rating Scale III (UPDRS III) was 62 (left: 25 right: 15) at the baseline, and 17 (left: 6, right: 3) at Med-On. He showed an improvement rate of 73%, following the acute levodopa challenge test (ALCT), and DBS was proposed to be performed. He reports no other neurological diseases, heart, liver, kidney, lung, or other major organ diseases, immune diseases, or allergies to medications or foods. However, the patient complained that a metal bullet was accidentally shot into the left brain from the edge of the left eye while playing with the pistol 40 years ago, and there was no other discomfort except blurred vision in the left eye.

### Neuroimage

**Figure 1:**
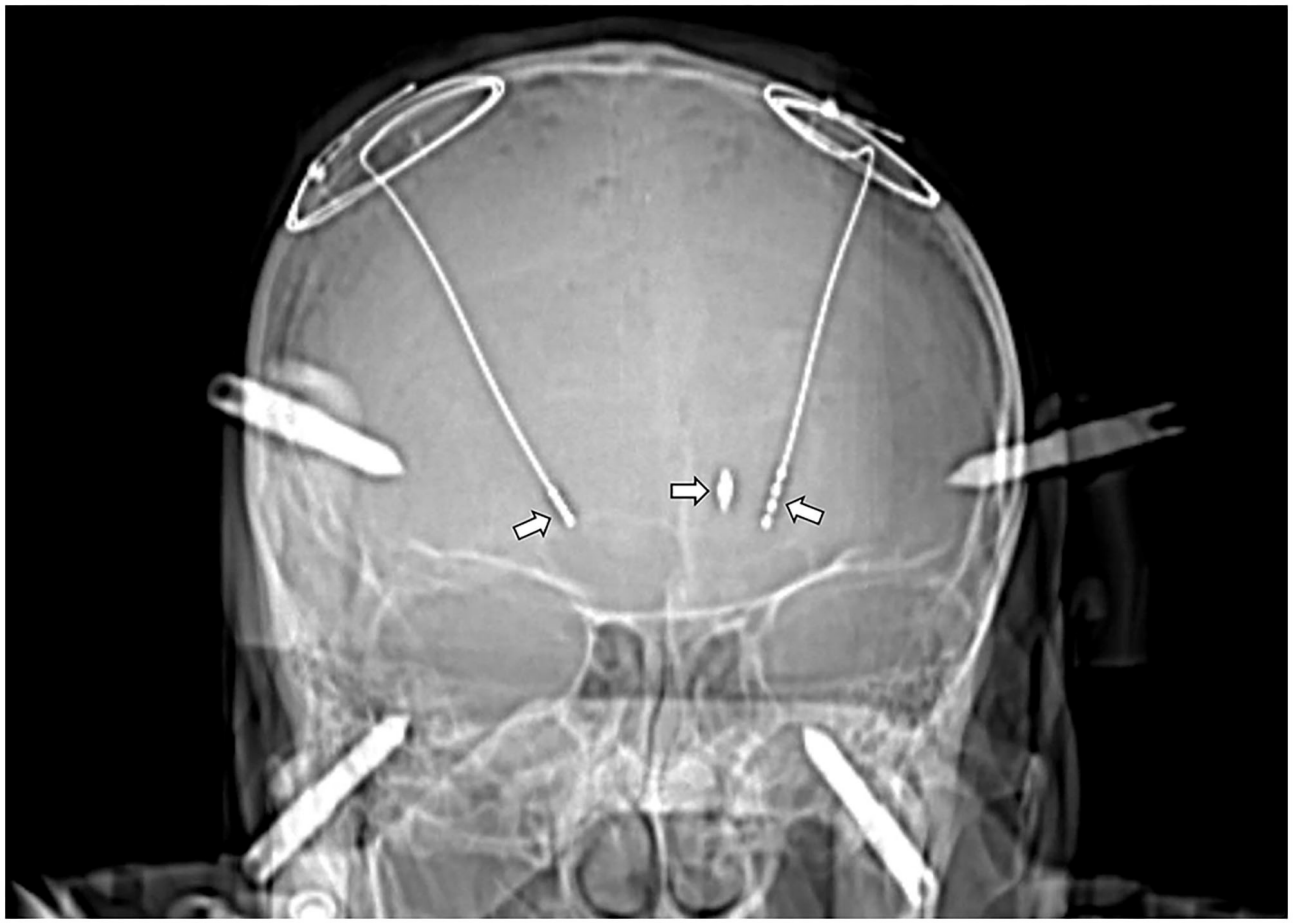
CT image after the DBS implantation (left arrow: STN-DBS implantation in the right cerebral hemisphere; right arrow: GPi-DBS implantation in the left cerebral hemisphere; mid arrow: The bullet was located on the left STN electrode trajectories).

**Figure 2:**
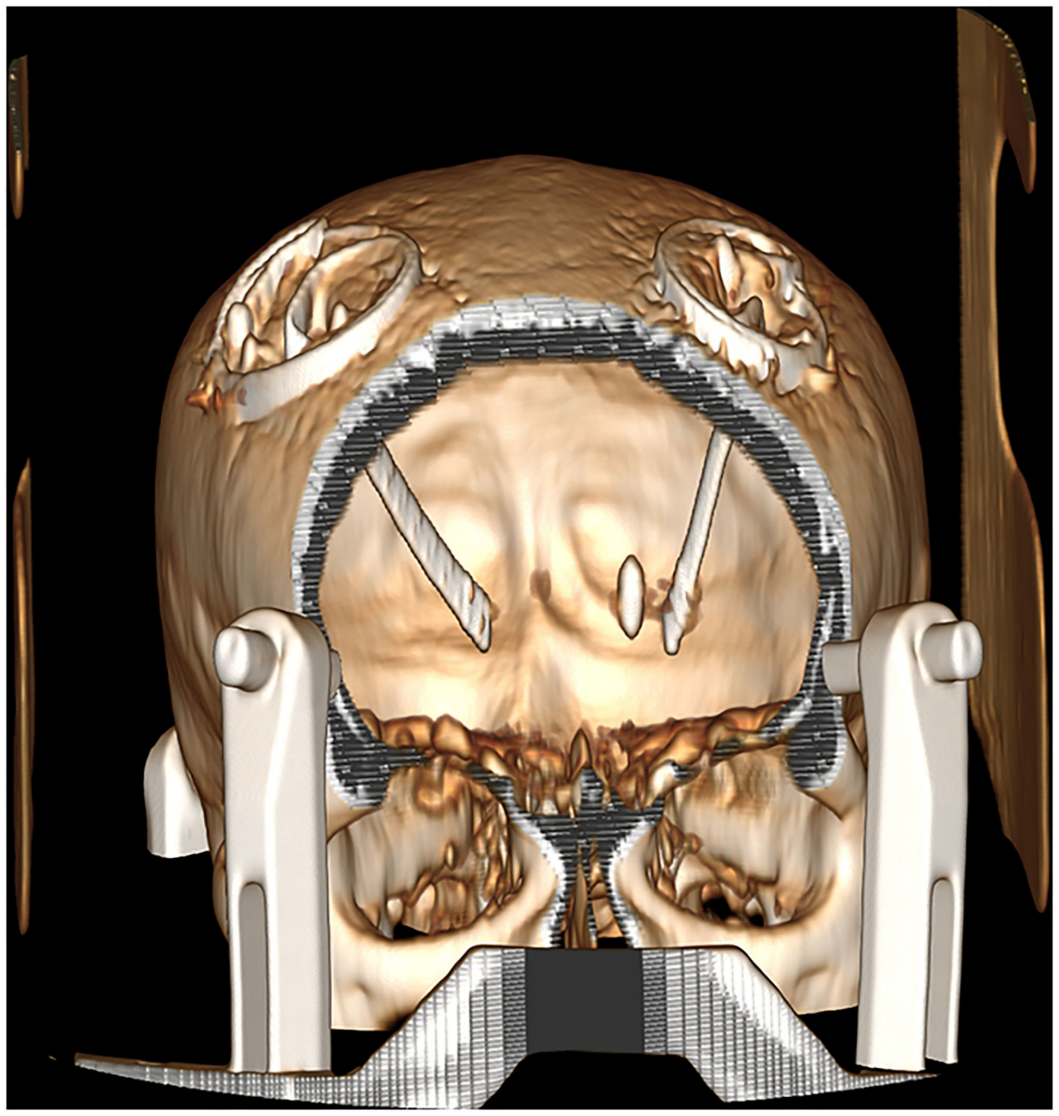
Three-dimensional reconstruction after the DBS implantation.

Due to the presence of a metal shrapnel foreign body in the patient's brain, MRI could not be performed. We can only use CT to determine the intracranial situation. The CT imaging demonstrates elderly brain changes with a dense metallic shadow in the left basal ganglia region, surrounded by radiating artifacts (Figures 1, 2).

### Operation

The results of the evaluation showed that the patient met the indications for DBS surgery and was proposed for DBS operation. Since the bullet was located on the left STN electrode trajectory, we gave the patient GPi-DBS implantation in the left hemisphere and STN-DBS implantation in the right hemisphere. Stereotactic CT images were acquired using a Leksell frame and coregistered with preoperative CT imaging. Planning the trajectory of the electrodes was done using the Medtronic Stealthstation S7 (Medtronic Inc., Minneapolis, MN). The target area was selected with GPI-DBS, avoiding the metal bullet path at the left cerebral hemisphere, and the right cerebral hemisphere was treated with STN-DBS. Since the target calculation was based on empirical target points from CT, so electrophysiological monitoring was strictly used to identify the functional regions of the nucleus. Lead point microelectrode recording system (MER) recorded the typical GPi signal on the left and STN signal on the right. After experimental intraoperative stimulation testing, the patient's associated tremor and stiffness symptoms were significantly relieved, and the increased voltage had no significant side effects. Final implantations were achieved by using leads (Medtronic Model 3387S lead at left and 3389S at right) and ACTIVA RC stimulator (Medtronic Inc., Minneapolis, MN). The dural burr hole was sealed with Porcine Fibrin Sealant Kit (Bioseal Biotech Co., Ltd. China). The surgery and postoperative course were uneventful, and the stimulator was not turned on immediately after the surgery.

### Postoperative Course

The patient was in good mental condition and could walk independently on the first postoperative day. The postoperative micro-lesioning effect was obvious with reduced shaking of the limbs. Computed tomography imaging showed that the electrode was accurately positioned with no intracranial abnormality. Furthermore, the stimulator was turned on 4 weeks after the operation, after the activation of left-brain GPI stimulation; the stiffness of the patient's right limb was significantly relieved; and, after the activation of right-brain STN stimulation, the tremor and stiffness of the patient's left limb were significantly relieved. The UPDRS III Scale was 8 (left: 2, right: 0) at Med-On and Stim-On (Stimulation improvement, 52% under Med-On). His tremor and stiffness symptoms were completely improved and could return to normal activities. We advised the patient to continue the preoperative anti-Parkinson medication regimen and timely follow-up. During the follow-up and review 6 months after the operation, we gave the patient DBS programming for one time, and the PD symptoms were well-controlled without no special discomfort. We recommend the patient to continue taking Madopar 125 mg q.i.d and withdrawal from Sifrol, periodic review of DBS program parameters, and further cranial imaging.

## Discussion

Parkinson's disease is a progressive neurodegenerative disease for which there is currently no cure and only a limited number of therapeutic options. Although the exact cause of PD is still unknown, it may be associated with a variety of factors, including aging, genetic susceptibility, and environmental exposures (Bloem et al., 2021). Traumatic brain injury (TBI) is one of the risk factors in PD (Jafari et al., 2013). Numerous studies have shown that TBI can trigger neuroinflammation (Qian et al., 2010), disrupt the blood-brain barrier (BBB), and result in leukocyte infiltration and microglial activation (Stoll et al., 2002). Additionally, TBI can impair mitochondrial function and result in glutamate excitotoxicity, both of which have been implicated in neurodegenerative diseases such as PD (Jafari et al., 2013). Additionally, elevated α-synuclein levels have been detected in the cerebrospinal fluid (CSF) of patients who have suffered a severe TBI (Mondello et al., 2013). A previous study indicated that patients who sustain a TBI late in life had a 44% increased risk of developing PD after 5–7 years (Gardner et al., 2015). Other studies have discovered that, except for TBI events occurring within 10 years before the diagnosis of PD, early TBI increases the risk of developing PD [the OR (odds ratio) for PD was 1.37 for every 5-year earlier age at first head injury with loss of consciousness] (Taylor et al., 2016). Despite recent research findings supporting the relationship between TBI and PD, the association remains somewhat contentious. First, comparisons between studies are complicated by differences in the timing, type, and extent of TBI. Then, because patients with PD are more likely to associate their disease with relevant trauma events, coupled with recall bias, the association between TBI and PD requires more in-depth and extensive investigation.

To the best of our knowledge, there have been only three cases worldwide in which patients developed movement disorders as a result of shrapnel retained in the brain, following a TBI: a 33-year-old male with Parkinson's syndrome secondary to shrapnel in the left midbrain (Shalash et al., 2018); a 38-year-old male with Parkinson's syndrome secondary to shrapnel in the left midbrain (Rondot et al., 1994); and a 20-year-old male with dystonia secondary to shrapnel in the left internal capsule (Polemikos et al., 2016). The three patients, however, experienced acute reactions and were not diagnosed with PD. In our case, the patient had a shot as a middle-aged adult 40 years ago (39-year-old) and developed PD 35 years later (74-year-old), during which time the patient had no neurological or psychiatric symptoms other than blurred vision in the left eye and no cranial trauma events were previously reported. Although research suggests a possible association between TBI and PD, the delayed onset of PD and absence of associated symptoms over 35 years make it difficult to consider gunshot wounds as a contributing factor to the onset of PD in this patient. Additionally, the onset was from the left limb, and the shrapnel lodged in the patient's left side of the brain. Further research is required to determine whether this laterality was caused by shrapnel in the left basal ganglia, limiting right side symptoms, or was unrelated to it. We did not perform a CSF or genetic screening on the patient for a variety of reasons, which limited our investigation of the etiology in this case.

Deep Brain Stimulation is one of the most effective methods for managing PD symptoms. Extensive long-term research on DBS target selection for PD has demonstrated that both GPi and STN are excellent targets for treating motor symptoms in patients with PD. STN has several advantages, including increased medication reduction, fewer battery adjustments, and a better economic profile. STN, on the other hand, does not affect axial symptoms, such as voice, swallowing, or balance. The advantages of GPi include ease of programming, great flexibility in medication adjustments (Williams et al., 2014), and a seemingly superior improvement in patients' axial symptoms such as gait (St George et al., 2010). There is currently no consensus regarding the appropriate target selection in patients with PD. In our case, the shrapnel was located on the left STN nuclear puncture path of the brain, which was difficult to avoid, and the patient's left limb tremor was obvious, while the right limb stiffness and other axial symptoms were severe (motor symptoms were obvious laterality). Therefore, we decided to perform bilateral DBS surgery on the patient, with GPi-DBS implantation in the left hemisphere and STN-DBS implantation in the right hemisphere. Although GPi-DBS implantation was performed in this case due to the presence of a metallic foreign body in the left STN electrode trajectory, this asymmetric targeting of the individualized and symptom-specific precision treatment protocol may relieve symptoms in patients with PD with asymmetric symptoms.

A review of one and a half years of postoperatively clinical curative effect in eight PD patients with asymmetry symptoms who were all treated with asymmetric target DBS surgery found that asymmetric target treatments had good clinical efficacy for both motor and non-motor symptoms of PD, as well as potential advantages in terms of axis symptoms, weight control, medication adjustment, and adverse side effects (Zhang et al., 2020). Therefore, asymmetric targeting treatments may combine the benefits of the two targets while avoiding the adverse effects associated with bilateral stimulation. Additionally, the flexibility and symptom-specific accuracy of asymmetric targeting protocols in clinical treatment are successful in this patient with PD with severe motor asymmetry, indicating that deeper investigation of this surgical treatment method is warranted.

To develop the puncture path, all that is currently required is coregistration of MRI and CT for nuclei target localization. However, due to the presence of metal shrapnel in the patient's brain, MRI could not be performed, making target calculation problematic with only CT imaging. The presence of intracerebral metal shrapnel artifacts and the interference of metal artifacts in the patient's head frame further complicated the design of the puncture path. Therefore, electrophysiological monitoring was strictly used to identify the functional regions of the nucleus because the target calculation was based on empirical target points from CT. Based on our experience, MER technology plays a critical role in nuclear localization in patients who are unable to undergo MRI examination.

The long-term effects of metallic foreign bodies in the brain on DBS electrical stimulation in terms of postoperative programming of patients have not been reported. Additionally, the relationship between the location and distance between metal foreign bodies and electrodes and the (side) effects of stimulation efficacy is unknown. In our case, the metal shrapnel in the brain was located in the posterior medial side of the left electrode at a distance of only 7 mm, which we believe could have influenced the electrical stimulation effect of DBS. As a result, we discovered that giving this electrode a smaller voltage parameter might have significantly alleviated the patient's motor symptoms, with fewer side effects and a larger therapeutic window of a voltage parameter, providing us with more programming flexibility. It was if the condition was caused by the patient's response to DBS or by the effects of the intracerebral shrapnel, which will require further research and long-term programming efficacy assessments.

## Conclusion

We report the first case of a patient with PD with the intracerebral metal shrapnel who was successful with DBS surgery. TBI may have been a risk factor in the occurrence and development of PD in the patient. The flexibility and symptom-specific precision of asymmetric targeting protocols in clinical treatment are effective in this patient with PD with severe motor asymmetry, and more exploration of this surgical treatment strategy is warranted. Additionally, MER technology is critical to nuclear localization in patients who are unable to undergo MRI examination. However, the long-term effects of metallic foreign bodies in the brain on DBS electrical stimulation are unknown, necessitating additional research and long-term monitoring.

## Data Availability Statement

The original contributions presented in the study are included in the article/supplementary material, further inquiries can be directed to the corresponding author/s.

## Ethics Statement

The studies involving human participants were reviewed and approved by First Affiliated Hospital of Xinjiang Medical University. The patients/participants provided their written informed consent to participate in this study. Written informed consent was obtained from the individual(s) for the publication of any potentially identifiable images or data included in this article.

## Author Contributions

YT and JW performed the image data process and wrote the manuscript. XS performed the target calculation, trajectory planning, and DBS parameter programming. ZF helped for giving the literature review. LJ contributed to perform the operation and give the manuscript preparation. YH helped perform the analysis with constructive discussions. All authors contributed to the article and approved the submitted version.

## Conflict of Interest

The authors declare that the research was conducted in the absence of any commercial or financial relationships that could be construed as a potential conflict of interest.

## Publisher's Note

All claims expressed in this article are solely those of the authors and do not necessarily represent those of their affiliated organizations, or those of the publisher, the editors and the reviewers. Any product that may be evaluated in this article, or claim that may be made by its manufacturer, is not guaranteed or endorsed by the publisher.
